# Rewriting Human History and Empowering Indigenous Communities with Genome Editing Tools

**DOI:** 10.3390/genes11010088

**Published:** 2020-01-12

**Authors:** Keolu Fox, Kartik Lakshmi Rallapalli, Alexis C. Komor

**Affiliations:** 1 Department of Anthropology, University of California, San Diego, La Jolla, CA 92093, USA; 2 Department of Global Health, University of California, San Diego, La Jolla, CA 92093, USA; 3 Department of Chemistry and Biochemistry, University of California, San Diego, La Jolla, CA 92093, USA; krallapa@ucsd.edu

**Keywords:** base editing, population genetics, signatures of natural selection, functional genomics

## Abstract

Appropriate empirical-based evidence and detailed theoretical considerations should be used for evolutionary explanations of phenotypic variation observed in the field of human population genetics (especially Indigenous populations). Investigators within the population genetics community frequently overlook the importance of these criteria when associating observed phenotypic variation with evolutionary explanations. A functional investigation of population-specific variation using cutting-edge genome editing tools has the potential to empower the population genetics community by holding “just-so” evolutionary explanations accountable. Here, we detail currently available precision genome editing tools and methods, with a particular emphasis on base editing, that can be applied to functionally investigate population-specific point mutations. We use the recent identification of thrifty mutations in the *CREBRF* gene as an example of the current dire need for an alliance between the fields of population genetics and genome editing.

## 1. Introduction

The development of next-generation human genome sequencing technologies has enabled the population genetics community to create large-scale databases of human genetic variation. Furthermore, the increasing accessibility of data analysis tools has resulted in geneticists better understanding the features of the human genome, including variable sites across the genome, chromosomal arrangements, and population-level variation (including the identification of population-specific point mutations). This pursuit of ever-increasing data has led to breakthroughs in ancestry assessments, multi-omic precision medicine models, and the identification of genetic risk factors [1]. However, while genome sequencing can be used to identify genetic variation associated with diseases, correlation does not equal causality.

Genome editing tools offer population geneticists the opportunity to conduct further assessment of the functional significance of population-specific variation derived from large-scale sequencing experiments. This has the potential to create accountability in the field of population genetics by functionally validating hypotheses surrounding genetic variation responsible for underlying traits or disorders. However, the use of genome editing technologies to study population-specific genetic variation is scant, particularly when compared to the number of studies on the use of genome editing for therapeutic purposes. Due to the ease with which researchers can now reprogram these tools for customized purposes (in large part thanks to the elucidation of the mechanics of CRISPR-Cas9 in 2012) [2], we believe now is the opportune time for a collaboration between the two fields. Moreover, the genome editing toolbox has rapidly broadened in recent years to include Cas variants with expanded editing scopes [3,4], high-fidelity variants [5,6], RNA editors [7,8], base editors [9,10,11], and, most recently, prime editors [12], which have collectively enabled researchers to modify the genomes of live cells with a higher precision, efficiency, and rapidity.

**Figure 1:**
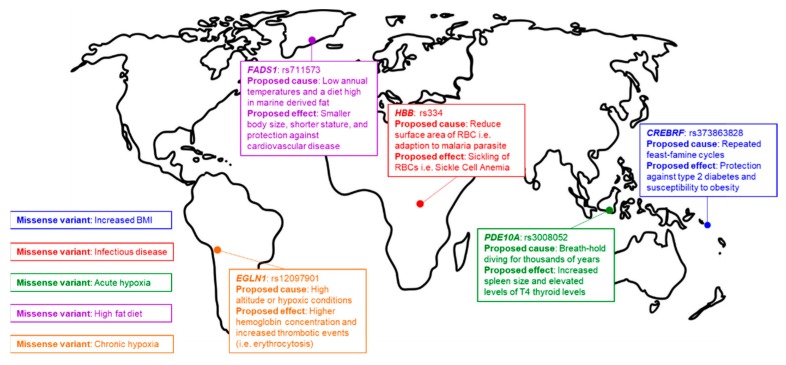
Signatures of natural selection. Examples of human local adaptations, each labeled by the phenotype and/or selection pressure, and the genetic loci under selection.

Point mutation introduction is of particular interest to the population genetics community, as many of the recently identified population-specific signatures of natural selection are single nucleotide variants (SNVs) [13]. In addition to understanding the effects of individual SNVs, we believe simultaneously introducing multiple mutations into the human genome in model systems via multiplexed editing will be essential for us to understand population-specific haplotypes, epistatic interactions, polygenic traits, and the effects that population-specific SNVs have on risk factor penetrance [14]. Specifically, SNV introduction tools can enable population geneticists to mechanistically connect genotype and phenotype, creating a new level of accountability in the identification of global signatures of human adaption and the narratives we build around signatures of natural selection (Figure 1). This perspective aims to describe the genome editing tools and methods, with a specific emphasis on base editing, that are currently available to equip population geneticists with the means to create accountability in the field and potentially empower Indigenous history. Furthermore, we hope to expose the genome editing field to the unique opportunity we have to functionally investigate signatures of natural selection in diverse human populations, using the “thrifty gene hypothesis” as an example [15].

## 2. Genome Editing Tools for Point Mutation Introduction

**Figure 2:**
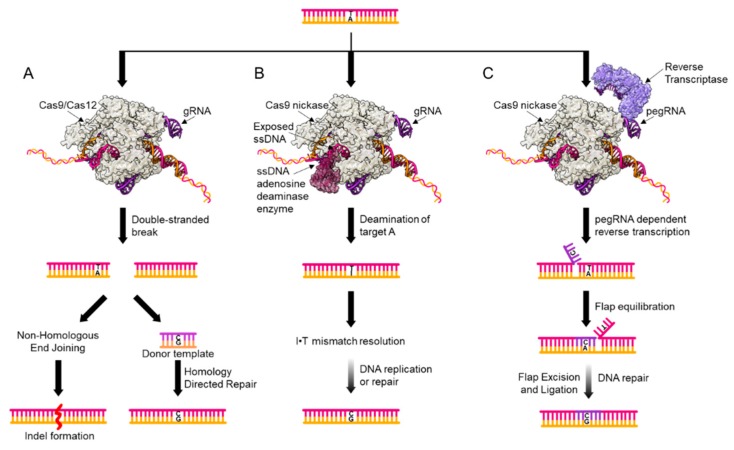
Genome editing tool kit. Currently available tools for point mutation introduction are shown, with a A•T to G•C shown as an example. (**A**) Double-stranded DNA break (DSB) introduction by RNA-guided Cas enzymes. The Cas endonuclease is programmed to bind and cut at a genomic locus of the researcher’s choosing by the sequence of the gRNA. Following DSB introduction, two repair pathways compete for resolution of the DSB, resulting in both indel formation and single nucleotide variant (SNV) introduction. (**B**) Base editors facilitate SNV introduction by chemically modifying a target DNA nucleobase into a uracil or inosine (inosine shown as an example). These non-canonical DNA bases are then processed by the cell, resulting in SNV introduction. (**C**) Prime editors use a 3′ extension on the gRNA as a template for a reverse transcriptase. Following the introduction of this sequence directly into the genome (light purple), flap resolution by cellular repair factors will remove the original genomic sequence to yield a mismatch, which is then processed by cellular repair factors to yield the SNV of interest.

The most established method for SNV introduction in mammalian cells relies on the initial introduction of a double-stranded DNA break (DSB) at a locus of interest. Currently, this is usually accomplished using CRISPR-Cas proteins (Figure 2A) [16]. In these systems, a Cas endonuclease (typically, a Cas9 or Cas12 variant) complexes with a piece of RNA (called a guide RNA or gRNA) that encodes the genomic coordinates at which the Cas:gRNA ribonucleoprotein (RNP) complex will bind. The RNP unwinds and binds to its target sequence (encoded by the sequence of the gRNA), forming an R-loop where one DNA strand is base-paired with the gRNA (the “target strand”), and another lacks a binding partner and is single-stranded (the “non-target strand”) [17]. Following DNA binding, the Cas enzyme will cleave the phosphodiester backbone of both DNA strands, resulting in a DSB that initiates the genome editing process. Once the DSB has been introduced, two DNA repair pathways compete to process the break. Processing by non-homologous end joining (NHEJ) will result in small base-pair insertions and deletions (indels) at the site of the DSB [18]. Researchers also have the option of providing the cell with an exogenous donor template which matches the DNA sequence surrounding the DSB, but encodes the SNV of interest. Homology-directed repair (HDR) can then use this template to replace a portion of the genome surrounding the DSB with the donor template sequence [19,20]. Genome editing via DSBs has been implemented since the mid 1990′s [21], making it the most well-studied and established mechanism for introducing point mutations into the genome of live mammalian cells.

An alternate approach for introducing certain types of point mutations is base editing (Figure 2B). This technology modifies the original CRISPR-Cas9 system by catalytically inactivating the endonuclease activity of Cas9 (to yield dCas9) and tethering it to an enzyme that chemically modifies cytosine nucleobases in a single-stranded DNA (ssDNA) context only (a cytosine deaminase enzyme). Upon R-loop formation, a small (~5 nucleotides when using the most common Cas9 variant) window of ssDNA is exposed, and the cytosine deaminase enzyme will convert any cytosines within this window to uracils (which have the hydrogen-bonding pattern of thymine). The resulting U•G mismatch is then processed by the cellular DNA replication and/or repair machinery to yield a T•A base-pair via a currently un-elucidated mechanism [10,11]. The original cytosine base editor (CBE), which catalyzes an overall C•G to T•A base-pair conversion, has been modified and updated extensively since its inception [22,23,24,25,26,27,28]. Current versions use a uracil glycosylase inhibitor (UGI) peptide to protect the uracil intermediate from excision by the endogenous DNA repair machinery, and a nickase version of Cas9 (Cas9n) that installs an ssDNA break in the DNA backbone of the strand opposite the uracil to bias repair outcomes in the researcher’s favor. Not long after the CBE’s introduction into the genome editing field, an adenine base editor (ABE) was engineered from a tRNA adenosine deaminase enzyme [9]. The ABE works analogously to CBE, but instead deaminates target adenosine nucleobases in the ssDNA window to inosines, resulting in an overall A•T to G•C base pair conversion, via an I•T intermediate. Due to the less toxic nature of these genome editing intermediates, base editors offer the community an opportunity to install multiple point mutations at a time, a capability that DSB-reliant tools lack. Base editing has been rapidly and enthusiastically adopted by the genome editing community. Despite its infancy, base editing has already been shown to be compatible with a wide range of organisms and cell types [29,30,31,32,33,34].

The most recent addition to the SNV introduction toolbox is prime editing (Figure 2C) [12]. This technology also co-opts the original CRISPR-Cas9 system, but in a different manner than base editing. Prime editors are comprised of a Cas9n fused to a reverse-transcriptase (RT) enzyme, and a modified, extended gRNA, called a pegRNA. This 3′ extension to the canonical gRNA serves as a priming element and encodes for the genomic modification of interest. Once the prime editor binds to its target locus, the Cas9n element cleaves the non-target strand, liberating half of the strand and allowing it to base-pair (or “prime”) to the 3′ extension of the pegRNA. The RT then recognizes this resulting DNA-RNA heteroduplex, and extends the DNA strand, using the pegRNA sequence as a template. Following the departure of the prime editor from the DNA, flap resolution by the endogenous DNA repair machinery (most likely by the enzyme FEN1) will result in a mismatch at the site of editing, which can be resolved by DNA replication or repair to install the point mutation of interest half of the time. To bias the resolution of the mismatch into the outcome of interest, an additional gRNA can be supplied to nick the strand harboring the original sequence. While it is too early for prime editing to have been adopted by the community, we anticipate that it will prove itself to be a powerful research tool.

## 3. Limitations of Current Tools

Genome editing via DSBs, while well-established, ubiquitous, and seemingly unrestrictive in the types of modifications with which it can be used to install, does suffer from certain downsides. Most importantly, NHEJ and HDR compete with one another to resolve the DSB intermediate, and therefore, many traditional genome editing experiments result in mixtures of genome-edited products. Indel formation is typically far more efficient than precision editing via HDR, particularly for point mutation introduction [16,35]. Furthermore, the HDR machinery is cell cycle-dependent, which has limited the types of cells amenable to precision genome editing by DSBs [36,37]. DSBs are a particularly toxic type of DNA damage and can result in a reduction in cell viability and even an enrichment for cells with p53 mutations [38,39]. Finally, attempts to multiplex editing via the introduction of multiple DSBs can result in large-scale chromosomal rearrangements [40]. A major research avenue in the field has therefore been to develop additional tools and methods to provide researchers with better control over genome editing outcomes, particularly with respect to point mutation introduction.

The main limitation of base editors is their inability to facilitate the introduction of point mutations beyond C•G to T•A or A•T to G•C. To overcome these limitations, future base editors will need to be developed that utilize new nucleic acid chemistries or creative DNA repair manipulation strategies. While the cellular DNA repair mechanisms by which base editors work have not yet been explicitly determined, point mutation introduction by base editing occurs with higher efficiencies and precision than that of HDR methods in most cases. However, cellular uracil excision by the base excision repair protein uracil-N-glycosylase is incredibly efficient and can cause C•G to non-T•A editing outcomes by CBEs in a locus-dependent manner. This can be combated by using CBE variants with optimized architectures and additional UGI components [23]. As their intermediates (uracil and inosine) are less cytotoxic than DSBs, and their point mutation introduction efficiencies are high (reaching >75% in many cases), base editors represent an exciting option for multiplexing, and we anticipate that base editors will significantly enable our ability to functionally investigate haplotypes in the future.

Base editors can also suffer from different types of off-target editing. It is well-documented that Cas9 can bind to sites in the genome that do not perfectly match the sequence of its gRNA. Base editors can introduce gRNA-dependent off-target point mutations at such sites in the genome [41,42]. These can be alleviated by using high-fidelity Cas variants [28]. However, in another form of gRNA-dependent off-targeting, which we call “bystander editing”, base editors can introduce multiple mutations at once at the on-target site. This is due to the processivity of the ssDNA modifying enzymes, which modify all cytosine or adenosines within the 5-nucleotide base editing window. Mutations can be installed into the deaminases to make them less processive, or impart upon them a sequence preference to reduce bystander mutations [25,43]. Additionally, a judicious choice of gRNA design can push these bystanders outside of the base editing window. Finally, base editors can introduce gRNA-independent off-target mutations, both in DNA (in the case of CBEs only) and RNA (both CBEs and ABEs) [44,45]. Mutations in the deaminases have been identified to eliminate off-target RNA editing [46,47,48], but have yet to be identified as eliminating off-target DNA editing by CBEs. We anticipate that this pressing issue will be resolved through future engineering efforts.

Prime editors are the most recent addition to the genome editing toolbox, and thus few experiments utilizing this technology are in print. In particular, little is known about the cellular repair mechanisms by which prime editing functions. The elucidation of these details will likely uncover what cell types are most (and least, if any) amenable to prime editing. While prime editors certainly outperform HDR-mediated editing to introduce point mutations, when using the nickase version of the technology, indel rates can reach levels around 10%, which can be too high for certain applications. Additionally, as with all newly developed genome editing agents, deep investigations into the scope of prime editing off-targets is required. Nevertheless, the technology is certainly exciting and has the potential to significantly aid researchers in a variety of applications.

## 4. Signatures of Natural Selection

The spread of human populations across the globe has led to genetic adaptations to diverse local environments (Figure 1). Recent developments in genomic technologies, statistical analyses, and expanded sampled populations have led to an improved identification and fine-mapping of genetic variants associated with adaptations to regional living conditions and dietary practices (i.e., signatures of natural selection) [13]. Ongoing efforts in sequencing genomes of indigenous populations, accompanied by the growing availability of “-omics” and ancient DNA data, promise a new era in our understanding of recent human evolution and the origins of variable traits and disease risks [49,50,51].

Many classic examples of signatures of natural selection that have defined our understanding of human adaptation have been discovered in the last century [13]. However, none have been more impactful than the discovery and mechanistic characterization of Sickle Cell Disease (SCD) as a result of the presence of infectious disease in equatorial African populations (i.e., the malaria parasite) [52,53]. SCD, almost exclusively identified in populations of African ancestry, is caused by an A•T to T•A point mutation in *HBB*, the b-globin gene [53].This results in a glutamic acid to valine substitution in hemoglobin S (referred to as HbS), creating a hydrophobic patch on the surface of the protein which causes the clumping of HbS molecules into rigid fibers and “sickling” of all the red blood cells in the homozygous (HbS/HbS) condition [54]. Importantly, the malaria parasite cannot reproduce using “sickled” red blood cells, which gives HbS carriers an adaptive advantage in areas with a high malaria incidence. Elucidation of the cause of SCD, and the realization that this disease has persisted in certain populations due to its protection against malaria, initiated a paradigm shift in the population genetics community—the evolutionary narratives that had been popularized in the field could be strengthened by connecting genotype to phenotype.

In the wake of the next-generation sequencing revolution, countless additional mutations associated with human adaptations to different environments have been identified (Figure 1) [55,56,57,58]. However, unlike SCD, most identified variants do not have straight-forward, biochemically-characterizable phenotypes. For example, mutations in *FADS1* are associated with a smaller body size and protection against cardiovascular disease in the indigenous Inuit populations of Greenland [56,59]. The requirement of large datasets in order to accurately associate genotype with phenotype further complicates the identification of signatures of natural selection in minority populations and indigenous people, as eighty-eight percent of large-scale screens of human genetic variation exclusively feature individuals of European ancestry [60]. This bias and systematic lack of engagement of underrepresented minorities and Indigenous people in genome studies can lead to the inaccurate interpretation of genome sequence data and the adoption of ”just-so” stories (overly simplified and unsubstantiated explanations of biological traits, behaviors, and practices) to explain evolutionary mechanisms [61].

Although controversial, one such example of a “just-so” story that affects an indigenous population is the “thrifty” missense variant rs373863828, p.(Arg457Gln), in *CREBRF* (a gene that encodes a regulator of CREB3, a transcription factor involved in inflammatory gene expression) [62]. This variant has been associated with a higher body mass index (BMI) per copy, with a ~1.3-fold greater risk of obesity in the Samoan population residing in Samoa and American Samoa [62,63]. The high minor allele frequency of the rs373863828 missense variant among Samoans in Samoa and American Samoa and in the Kingdom of Tonga [64], compared to an exceedingly rare frequency in other populations in the Genome Aggregation Database [65], supports the hypothesis that this variant is an important risk factor for obesity unique to the Samoan and Tongan populations and possibly other Polynesian populations. However, unlike many other obesity risk variants in other populations, this BMI-increasing allele has been associated with a lower odds of type 2 diabetes (T2D) [63,66]. Clearly, a functional investigation of the variant and its mechanism of action is required and has the potential to improve minority health disparities.

## 5. The “Thrifty Gene Hypothesis”

Evolutionary models have been proposed to explain the high prevalence of metabolic disease in modern populations [67]. The best-known model to explain predilection to metabolic disease is the “thrifty genotype hypothesis”. The thrifty gene model, first proposed by James V. Neel in 1962, is an attempt to explain the existence of diabetes susceptibility alleles in modern populations [15]. These so-called thrifty genes enable individuals to efficiently store energy as fat to sustain themselves during periods of famine. Consequently, during periods of food abundance, these variants result in obesity. The thrifty genotype hypothesis has been widely used to explain the high incidences of metabolic disease among westernized Native Americans, Australian Aborigines, and Pacific Islander populations [62,68]. It is argued that, because these regions of the world were settled under potentially food-scarce circumstances, the thrifty genotype was strongly favored [69]. However, it seems likely that food shortages were an ever-present threat for all our ancestors and unlikely that this threat suddenly disappeared with the advent of agriculture [70]. Furthermore, paleopathological evidence suggests that nutrition among early farmers was often poorer than among hunter-gatherers [71], which would have caused the thrifty genotype to be favorable in wider group populations until very recently (i.e., the post-industrial revolution) [72].

Despite many critiques of the thrifty gene hypothesis [69,73,74,75] (Sellayah, Cagampang, and Cox 2014), and scant genetic evidence in support of the thrifty hypothesis (i.e., the detection of selection signatures in genes involved in metabolism) (Helgason et al. 2007) (Ayub et al. 2014) (Steinthorsdottir et al. 2014), the thrifty genotype hypothesis continues to be utilized as an explanation for disparities in metabolic health in developing nations [62,75]. This is despite the ground-breaking publication by Gould and Lewontin, which cautioned against the use of evolutionary explanations for phenotypic variation without (1) appropriate empirical-based evidence and (2) detailed theoretical considerations [61]. Investigators today frequently overlook the importance of these criteria when associating observed phenotypic variation with evolutionary explanations [76]. Specifically, regarding the settlement of Polynesia, ethnographic, archeological, and paleoethnobotanical evidence does not support long periods of famine while voyaging in search of far-flung island archipelagos in the Pacific Ocean [77,78,79]. On the contrary, ethnographic, archeological, and paleoethnobotanical evidence supports the utilization of advanced horticultural methods and wayfaring technology providing an excess of calories while people populated various island land masses throughout Austronesia [78,79].

Importantly, the thrifty gene hypothesis is problematic from a colonial perspective in that it discredits the way-finding capabilities of voyaging societies [79]. Furthermore, in its failure to properly identify the evolutionary forces and biochemical mechanisms responsible for the high rates of metabolic disease observed in Native Americans, Australian Aborigines, and Pacific Islander populations, the thrifty gene hypothesis indirectly complicates minority health disparities [80]. It is far more likely that the introduction of leprosy, smallpox, syphilis, and other diseases from European colonialists in the eighteenth-century lead to the “selective sweep” (i.e., a population bottleneck) that is observed in modern Polynesian populations today [62,81], particularly given *CREBRF*’s involvement in the regulation of genes involved in inflammation [82]. Through a collaboration between the fields of population genetics and genome editing, we can begin to test these hypotheses in model systems (i.e., observe if thrifty variants impart a selective advantage against nutrient deprivation or exposure to diseases), and understand the underlying mechanisms behind obesity risk variants to create treatment options for at-risk communities.

## 6. Previous Methods for Functional Investigation

While the identification of this so-called thrifty mutation is compelling, metabolic disease is epistatic, involving complex networks of interactions [82]. Indeed, many of the population-specific variants identified to-date are implicated with complex traits or disorders (Figure 1). Understanding and elucidating the functional consequence of these variants therefore represents a daunting task [83]. Classical genetic approaches for interpreting variation, such as case-control or co-segregation studies, require the identification of many individuals with each variant [84]. However, this strategy has clear limitations for studying variation found in minority populations [85]. Fully realizing the clinical potential of genetics requires accurately inferring pathogenicity, even for rare or private/population-specific variation [1]. Many computational approaches to predicting variant effects have been developed, but they can only identify a small fraction of pathogenic variants with a high confidence [86,87]. There is therefore an undeniable need for cellular and in vivo studies to functionally validate population-specific variation [1,88,89,90].

Experimentally measuring a variant’s functional consequences can provide clearer guidance. One method for studying the effects that mutations have on protein function is in vitro experiments performed with purified protein. However, many population-specific variants of interest occur in genes encoding for proteins that act as part of a variety of pathways and interact with many other proteins in their native context. Therefore, in vitro assays that study them in isolation outside of the cellular environment will miss important effects due to interactions with other biomolecules within the cell. A more popular method that has been employed previously is to knock-out the endogenous gene and supply the cell with an exogenous copy harboring the variant of interest. However, this often results in overexpression of the protein of interest, which can convolute the interpretation of data [62]. Importantly, neither of these two methods are capable of investigating non-coding variants, including those found in intronic regions, enhancers, and long non-coding RNAs. Primary cells taken from individuals with and without a variant of interest have also been used in the past to study the functional consequences of genetic variants, but genetic variation of the individuals at other locations in the genome can introduce confounding variables that invalidate direct comparisons of the two cell lines.

Genome editing can be used to introduce mutations of interest directly into the endogenous locus of living cells in an appropriate cellular or in vivo model system. This allows researchers to study the functional consequences of a variant of interest in the most physiologically relevant context, and allows for direct comparisons with wild-types with no genetic background variation issues. The generation of isogenic cell lines (cell lines with completely matched genotypes except for a variant of interest) harboring population-specific variants will allow us to study a variety of minority health-related SNVs in a meaningful manner. However, generating these cell lines using traditional, DSB-reliant methods has proven cost- and time-prohibitive due to the low point mutation introduction efficiencies and the high indel levels. Typically, researchers must screen ~500 clones to obtain a single heterozygous mutant, which represents a significant roadblock to many labs [91]. Fortunately, base editors and prime editors introduce SNVs with significantly higher SNV:indel ratios, offering a promising alternative in generating models of population-specific diseases [92]. Furthermore, as our understanding of human genetics expands, we predict that the need to study epistatic traits and haplotypes (i.e., multiple SNVs at a time) will be necessary to fully understand the relationship between genetic variation and traits and disease. Base editors and prime editors offer scientists the tools to introduce multiple variants at a time and will thus prove themselves to be instrumental in understanding genetic variation. This “reverse genetics” approach not only has immense potential for future functional assessments for variation that is computationally identified as pathogenic or under selection, but the potential for personalized treatment of metabolic disease in Polynesian populations.

Finally, we recognize that environmental interactions in laboratory settings do not fully recapitulate natural environments or environments of the past. As a result, we recognize that gene–environment interaction(s) is an important variable to consider when attempting to understand the relationship between genotype and phenotype—especially in underrepresented Indigenous populations (both modern and ancient). While reverse engineering via precision genome-editing tools offers an interesting preliminary perspective on the mechanistic function of population-specific heterogeneity in vitro, another avenue for innovation is reverse engineering the environment or selective pressure itself (e.g., simulating or adjusting the amount of oxygen in an environment where physiologically relevant cells are incubated to simulate the hypoxic environment of Himalayan and Andean indigenous populations) [93,94]. Alternatively, similar experiments could be conducted at a high elevation in hypoxic environments (i.e., Indigenous-operated laboratories in either the Himalayas or Andes).

## 7. Future Prospects

For the entirety of human history, diverse ecological pressure has shaped the genomes of indigenous populations around the world, resulting in phenotypic diversity and signatures of natural selection. While this variation once provided these individuals with a selective advantage over their peers, in the present day, this is not always the case. Specifically, modern Polynesian populations display some of the highest rates of metabolic disease observed globally due to this phenomenon. Functionally investigating the mechanism and cause of metabolic disease susceptibility in Polynesia not only allows the population genetics community to test “just-so” stories, but also has the potential to lead to disease prediction, prevention, and the design of personalized treatment for metabolic disease in the future.

While there is tremendous potential for precision genome editing tools to empower Indigenous communities through medical actionability and repatriating our deep past, it is also important to think about who uses these tools. For example, Indigenous researchers familiar with the history of the populations they are partnering with are unlikely to discount the knowledge and capabilities of their ancestors [95]. Moreover, Indigenous researchers familiar with the communities they are partnering with are better positioned to use genome editing tools to predict and prevent disease in their communities [95].

Currently, 95% of clinical trials in the United States have included individuals of European ancestry, yet underrepresented minority populations such as Polynesians face significantly higher rates of common complex diseases (e.g., cancer, cardiovascular, and metabolic disease) [96]. Insights from studies that include minority populations should have benefits that yield modern medical treatments, potentially reducing the widening gap observed in health disparities [97,98].

Finally, it is our responsibility as members of the genome engineering and population genetics community to identify potential profiteers and ensure that biomedical patents do not create conflicts between potential stakeholders in ways that other patents typically do [99]. Given the history of exploitation of Indigenous peoples in biomedical research, it is crucial that we build equitable partnerships with Indigenous communities. By involving Indigenous peoples in the research surrounding genetic variation identified in their communities, we can engage in more ethical and equitable research and encourage Indigenous self-governance in biomedical research [95].

As next generation sequencing and genome editing tools become more ubiquitous, diverse communities are included in large-scale surveys of human genetic variation [100], and population-specific reference genomes are generated, the population genetics community will have the potential to identify additional signatures of natural selection (both single-nucleotide and structural variants) in known candidate genes, overlooked pathways [101], and novel genes to uncover putative causes of disease in marginalized populations [102]. This will allow us to not only refine our understanding of precision medicine in the future [103], but also utilize emerging genomic technologies to create accountability in human population genetics and redefine understanding of our deep-history as a species and our relationship to our ancient ancestors.
